# Improvement of Iron and β‐Carotene Bioaccessibility in Complementary Foods: Biofortification of Local Crops With Organic Residual Products and Microorganisms

**DOI:** 10.1002/fsn3.4745

**Published:** 2025-01-19

**Authors:** Mbeugué Thiam, Adama Diouf, Christèle Icard‐Vernière, Sylvie Avallone, Ndèye Fatou Ndiaye, Marielle Atala De Souza, Jean‐Michel Médoc, Nicole Idohou‐Dossou, Christèle Humblot

**Affiliations:** ^1^ Laboratoire de Recherche en Nutrition et Alimentation Humaine, Faculté des Sciences et Techniques Université Cheikh Anta Diop (UCAD) Dakar‐Fann BP Senegal; ^2^ UMR Qualisud, Univ. Montpellier, Avignon Université, CIRAD, Institut Agro, IRD, Université de la Réunion Montpellier France; ^3^ Institut de Technologies Alimentaires (ITA) Dakar Hann Senegal; ^4^ CIRAD UPR Recyclage et Risque Montpellier France; ^5^ CIRAD Recyclage et Risque, Univ. Montpellier Montpellier France

**Keywords:** agronomic biofortification, bioaccessibility, complementary foods, micronutrient deficiencies

## Abstract

Micronutrient deficiencies remain a great public health challenge worldwide with iron, zinc, and vitamin A being the most problematic. It has been shown that biofortification through agronomic strategies can increase their micronutrient content, but data on the bioavailability remain limited. In Senegal, consumption of cereals and legumes is high, and orange‐fleshed sweet potato (OFSP), rich in β‐carotene, has been introduced a decade ago. The objective of the present work was to assess the bioaccessibility of iron, zinc, and β‐carotene in local complementary foods prepared with millet, cowpea, and OFSP alone or in combination, produced using different agronomic biofortification strategies. Organic residual products were used alone or in combination with microorganisms to produce the abovementioned crops that were used to prepare the complementary foods. Static in vitro digestion was performed to assess the bioaccessibility of the micronutrients, according to a harmonized protocol. The two organic residual products had different effect, as the cow dung alone was inefficient to increase iron and zinc contents as well as their bioaccessibility in millet porridges. However, the use of poultry litter alone or in combination with microorganisms increased iron bioaccessibility in cooked cowpea (27%–29%) compared to the non‐biofortified counterpart (9%). Surprisingly, bioaccessible β‐carotene was significantly higher (4.1%) in sample of mashed OFSP biofortified with the combination of the different agronomic strategies than in the others (1.4%–2.5%). Portions (150 g) of porridge prepared from the three biofortified crops would cover up to 100% of the daily vitamin A requirements of children aged 6–23 months. The use of a combination the most promising varieties of crops, together with the agronomic strategies, would be a complementary approach to sustainability limit micronutrient deficiencies in a context of monotonous diets.

## Graphical abstract

Micronutrient deficiencies remain a great public health challenge worldwide with iron, zinc, and vitamin A being the most problematic. It has been shown that biofortification through agronomic strategies can increase their micronutrient content, but data on the bioavailability remain limited. This study confirms that the combination of the most promising varieties of crops, together with the agronomic strategies, would be a complementary approach to sustainability limit micronutrient deficiencies using local complementary foods in a context of monotonous diets.

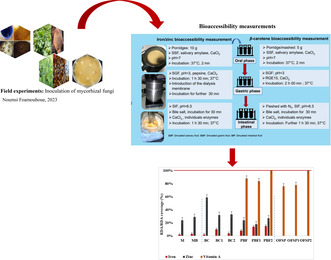

## 1 Introduction

Senegal is one of the sub‐Saharan African countries that still face multiple malnutrition issues, despite several strategies implemented over decades. In the rural settings of the groundnut basin (one of the agroecological regions of Senegal), particularly in the Kaffrine region, the persistence of wasting and stunting in children under 5 years and the presence of the double burden of malnutrition in women of reproductive age (WRA) were recently highlighted. Indeed, 15.7% of children under 5 years suffer from wasting and 26% suffer from stunting (COSFAM 2022); while more than 20% of WRA are wasted or overweight/obese (Thiam et al. 2022). Concerning hidden hunger, in rural areas, iron deficiency affects 61.5% of children aged 12–59 months (COSFAM 2022). Among WRA, more than 30% are anemic and 40% are iron deficient (Badiane 2018; ITA, COSFAM, and NI 2022). As reported in many other works, malnutrition in all its forms increases intra uterine growth retardation, resulting in low birth weight, impaired psychomotor and cognitive development, lower school performances, and then in adulthood increase non‐communicable diseases such as type 2 diabetes and high blood pressure (WHO 2002; Black et al. 2013; Victoria et al. 2016).

In the groundnut basin of Senegal, malnutrition issues are linked to nutrient inadequacy associated with monotonous dietary patterns, and feeding practices that are inappropriate for infants and young children (Thiam et al. 2022). In fact, the groundnut basin is the scene of extensive production and consumption of cereals (millet, maize) and cowpea. The population consume millet at least twice a day, at breakfast and dinner. Cowpea is an ingredient in many widely consumed traditional dishes. In addition to these monotonous diets, the scarce consumption of micronutrient‐rich foods, such as animal‐based products (meat, eggs, liver, and other organ meats) and fruits, due to household poverty, seasonality, as well as cultural norms and beliefs related to food taboos limits nutrient intake and increases the risk of iron, zinc, and vitamin A deficiencies. To fight against maternal and child malnutrition during the 1000 first days of life and vitamin A deficiency, orange‐fleshed sweet potato (OFSP) was introduced in this area in 2015 in a research project for development (Badiane 2018). Nevertheless, the issues of malnutrition still persist. The improvement of the nutritional quality of staple foods such as millet and cowpea consumed widely, as well as the promotion of OFSP, could be a way to prevent maternal and child malnutrition.

Agronomic biofortification involving the use of organic residual products (ORPs), mycorrhizal fungi (MF), and efficient microorganisms (EMs) could be an effective way to improve nutrient uptake from soils to plants and for human consumption (Khan et al. 2020; Teklu et al. 2023). A limited number of different studies have demonstrated the positive impact of biofortification in human nutrition through clinical trials which are gold standard methods in the field of nutrition (de Brauwn et al. 2015; Finkelstein et al. 2015; Haas et al. 2016; Malézieux et al. 2023). However, clinical trials are expensive, invasive, and do not always provide the expected physiological information (Mackie, Mulet‐Caberob, and Amelia Torcello‐Gómez 2020). In vitro methods are appealing alternatives to in vivo studies in humans and animals (Sulaiman, Givens, and Anitha 2021). Especially when the bioaccessibility of micronutrients needs to be assessed in different types of foods, in vitro methods are useful to simulate human digestion to limit human studies to the most promising foods. The assessment of nutrient bioaccessibility is the starting point to estimate the beneficial effects of biofortified products on human health (D'Imperio et al. 2022). The roles of ORP, EM, and MF in enhancing the solubilization of minerals (e.g., iron, zinc, Ca) and their availability in some plants like millet are well known to agronomists (Mangueze et al. 2018; Gonzalez et al. 2019; Montoya et al. 2020; Khan, Shah, and Tian 2022; Szerement et al. 2022; Teklu et al. 2023).

The present study is part of the OR4FOOD “Organic Residual products For Biofortified Food for Africa” project that aims to improve the micronutrient status of rural populations and to fight hidden hunger. For this purpose, in Senegal, agronomists, food technologists, microbiologists, and nutritionists have worked together to propose accessible African local foods biofortified in provitamin A, iron (Fe), and zinc (Zn) through agroecological practices focusing on the use of ORP, EM, and MF (Noumsi Foamouhoue 2023). Millet ( *Pennisetum glaucum* ), cowpea ( *Vigna unguiculata* [L.] Walp), and OFSP ( *Ipomoea batatas* [L.] Lam) were the local foods that were targeted due to their wide consumption and consumer acceptance. First, 10 varieties of each crop were selected for screening. Among the 10 varieties, two varieties of each crop were chosen based on their micronutrient content, yield per hectare, and mycorrhizal response (Diallo B et Founoune H, unpublished data). The selected varieties were then fertilized with different ORPs, namely, cow dung (CD, for millet only), poultry litter (PL), or PL + MF + EM (for cowpea and OFSP) to increase iron, zinc, and β‐carotene contents in the edible parts of the crops (Noumsi Foamouhoue 2023). Subsequently, the Institute of Food and Technology (ITA) of Senegal produced millet flours and blended flours composed of millet (*SL423* variety: 50%), cowpea (*Thieye* variety: 25%), and OFSP (*Apomuden* variety: 25%). The objective of the present work was to assess the effect of biofortification on iron, zinc, and β‐carotene bioaccessibility in local complementary foods prepared with millet, cowpea, and OFSP alone or in combination.

## 2 Materials and Methods

### 2.1 Reagents

Static in vitro digestion was performed using the following reagents: salivary α‐amylase (A 3403‐500 KU; Sigma), gastric lipase (RGE15; Lipolytech), pepsin from porcine gastric mucosa (P7000; Sigma), trypsin (T0303; Sigma), chymotrypsin (C4129; Sigma), amylase from porcine pancreas (A3176; Sigma), pancreatic lipase (L3136; Sigma), colipase (C3028; Sigma), bovine, and ovine bile salts (B8381; Sigma).

### 2.2 Enzyme Activity Assays and Simulated Digestive Fluids Preparation

Enzyme activity assays and simulated digestive fluids (oral, gastric, and intestinal) were performed using the methods described in the harmonized protocol (Brodkorb et al. 2019).

### 2.3 Food Samples

“As eaten” food samples were prepared from the abovementioned flours, cowpea grains, and OFSP tubers produced as part of the OR4FOOD project. They were cooked as follows:

- Millet porridges were prepared from non‐biofortified millet flour (M) and biofortified millet flour (MB). Each type of flour (50 g) was mixed with a sufficient quantity of water (SQW) in a vessel. The mixture was then poured into boiling water (100°C) and stirred for 7 min.
- Non‐biofortified cowpea (BC) and cowpea biofortified with PL (BC1) or PL + EM + MF (BC2) were soaked for 14 h and boiled at 100°C for 45 min. The three kinds of cowpeas were then blended separately in a blender until homogenized. Non‐biofortified OFSP and OFSP biofortified with PL (OFSP1) or PL + EM + MF (OFSP2) were rinsed, steamed for 30 min, peeled, and mashed using an Ultra‐Turrax homogenizer (T25 Werke; GmbH, Germany).
- Porridges made from blended flours (PBFs) were prepared from non‐biofortified mixed flours (PBF) and mixed flours composed of millet biofortified with CD (only) and cowpea + OFSP biofortified with PL (PBF1) or PL + EM + MF (PBF2). They were cooked in the same way as the millet porridges, except that the temperature was kept below 100°C (69°C–75.5°C) to avoid carotenoid degradation in the OFSP.

After cooking, non‐vitamin A–enriched oil (66 μL/g of food) was added to all mashed OFSP and PBFs to optimize micellarization of carotenoids and the bioaccessibility of β‐carotene (Sriwichai et al. 2016). To avoid iron and zinc contamination from exogenous sources, all the equipments were soaked in 15% nitric acid solution (408071 Carlo Erba) for 24 h, rinsed with distilled water (3 times) and ultrapure water (3 times). Titratable acidity of millet porridges, PBFs, and boiled cowpeas was determined according to Wolfgor et al. (2002) to compute the concentration of the PIPES buffer needed to keep the pH at 6.5 during intestinal digestion.

### 2.4 Measurement of Iron and Zinc Bioaccessibility

The bioaccessibility of iron and zinc was measured in the following food samples: millet porridges (*n* = 2), boiled cowpeas (*n* = 3), and PBFs (*n* = 3). In vitro digestion of each food sample was performed in triplicate. All the volumes of simulated digestive fluids, enzymes, and bile solutions required were determined using the online digestion spreadsheet of INFOGEST (Brodkorb et al. 2019). To simulate the oral phase, 10 g of each food sample (×3) were diluted with 8 mL of prewarmed (37°C) simulated salivary fluid (SSF) in 125‐mL glass tubes. Salivary amylase solution (1 mL) was added to achieve an activity of 75 U/mL in the final digestion mixture. After adding the CaCl_2_ (50 μL), the pH was adjusted to 7 with 1 M NaOH solution and samples were incubated horizontally in a shaking water bath (VWR VL53 EC; Memmert GmbH, Germany) at 37°C for 2 min. For gastric digestion, 16 mL of prewarmed simulated gastric fluid (SGF) were added in the oral bolus and the pH was adjusted to 3 with 6 M HCl solution. Pepsin (2000 U/mL of activity in the final digestion mixture) and CaCl_2_ (10 μL) solutions were added in each sample, the volume in each tube was completed to 40 mL with milli‐Q water and the samples were incubated at 37°C for 1 h 30 min. To start intestinal digestion, gastric chyme was diluted with 17 mL of prewarmed simulated intestinal fluid (SIF) and pH was adjusted to 6.5. Dialyzable iron and zinc were estimated using a dialysis membrane tubing (Spectra/Por4 Standard RC, average flat width 32 mm, molecular weight cut‐off 12–14 kD) containing 10 mL of 1,4‐piperazinediethanesulfonic acid disodium salt buffer (PIPES, pH 6.5, P3768; Sigma). Dialysis tubing containing 10 mL of PIPES buffer (pH = 6.5) and bile solution (5 mL) was added in each sample following by 30 min of incubation at 37°C for the complete solubilization of the bile. Finally, CaCl_2_ solution (80 μL) and 2 mL of each individual enzyme solution, namely trypsin (100 U/mL), chymotrypsin (25 U/mL), amylase from porcine pancreas (200 U/mL), lipase from porcine pancreas (2000 U/mL) were added. After adding milli‐Q water to complete the volume of tubes to 80 mL, the samples were again incubated for 1 h 30 min. At the end of the intestinal digestion, the dialysis tubing was removed and rinsed with milli‐Qwater and the dialysates were carefully poured into new conical tubes. Digestates (20 g) were centrifuged at 4500 rpm (Heraeus multifuge X1R, Cat. 75004250, Germany) for 45 min at 4°C to separate soluble and insoluble iron/zinc contained in the supernatant and in the pellets, respectively. Minerals contained in the samples (as eaten, digestates, dialysates, supernatants, and pellets) were extracted using a microwave digestion system (ETHOS‐Easy, Milestone, Italy; Berghof SW‐2 Harretstrasse 1, Germany) with 1 mL of 30% hydrogen peroxide (412072 Carlo Erba) and 7 mL of 69.5% nitric acid (408071 Carlo Erba). Dry matter (DM) was obtained by drying at 105°C in a JP Selecta 2000210 oven (Memmert 854 Schwabach, Germany) for 24 h. Iron and zinc contents in the millet porridges were analyzed with inductively coupled plasma‐optical emission spectrometry (ICP‐OES 5100; Agilent Technologies). For cowpea and PBFs, analyses were performed using an atomic absorption spectrometer (AAS; Perkin ElmerAAnalyst 800, USA). Bioaccessibility refers to the amount of a nutrient/compound that is release from a food matrix/product after digestion and potentially available for absorption through the gut epithelium. After digestion, either solubility or dialyzability are employed to assess the proportion of nutrient available for absorption. We used dialyzability for iron and zinc estimation of bioaccessibility since it is considered as more accurate to predict the bioavailability of the minerals (Fairweather‐Tait et al. 2007). Dialyzable, soluble non‐dialyzable (SND), and insoluble iron and zinc were computed according to Wolfgor et al. (2002).

$$\begin{array}{c} \text{Dialyzable iron or}\;\mathrm{Zn}\%\\ =C_{D}\;\left(W_{D}+W_{S}\right)/\left(C_{D}W_{D}+C_{S}W_{S}+C_{I}W_{I}\right)\times 100 \end{array}$$

$$\begin{array}{c} \text{Soluble non}-\text{dialyzable}\;\left(\mathrm{SND}\right)\;\text{iron or}\;\mathrm{Zn}\%\\ =W_{S}\;\left(C_{S}-C_{D}\right)/\left(C_{D}W_{D}+C_{S}W_{S}+C_{I}W_{I}\right)\times 100 \end{array}$$

$$\begin{array}{c} \text{Insoluble iron or}\;\mathrm{Zn}\%\\ =W_{I}\;\left(C_{I}\right)/\left(C_{D}W_{D}+C_{S}W_{S}+C_{I}W_{I}\right)\times 100 \end{array}$$

where *C*_D_, *C*_S_, and *C*_I_ are iron concentrations (μg per 100 g) and *W*_D_, *W*_S_, and *W*_I_ are the weights (g) of the dialysate, supernatant, and pellet, respectively.

### 2.5 Measurement of β‐Carotene Bioaccessibility

Bioaccessibility of β‐carotene was measured by gastrointestinal digestion performed with 5 g (×3) for each food sample (PBFs and mashed OFSP). Samples were first homogenized using an IKA Ultra‐Turrax (T25 Werke; GmbH, Germany) and 4 mL, 0.5 mL, and 25 μL of SSF, salivary amylase and CaCl_2_ solutions, respectively, were added. After adjusting the pH to 7, the final volume of each tube was completed to 10 mL with Milli‐Qwater and samples were then incubated at 37°C for 2 min in a shaking water bath. To mimic the gastric phase, 8 mL of SGF were added to the oral bolus and the pH was then adjusted to 3. Gastric extract solution (0.5 mL) and pepsin (0.5 mL) were poured into the samples to reach 60 U/mL for lipase and 2000 U/mL for pepsin in the final digestion mixture. After adding the CaCl_2_ solution (5 μL) and adjusting the final volume to 20 mL, the samples were incubated for 2 h. Prior to the small intestinal phase, the samples were flushed with azote (N_2_) for a few minutes to remove oxygen. Next, 8.5 mL of SIF were added and the pH was adjusted to 6.5. Bile solution (2.5 mL) was added in the samples and tubes were incubated for 30 min. Next, CaCl_2_ (40 μL) and 1 mL of each individuals pancreatic enzyme (trypsin, chymotrypsin, pancreatic amylase, pancreatic lipase, and colipase) were poured in samples. The tubes were then incubated at 37°C for 1 h 30 min. The amount of solubilized β‐carotene was chosen as a measure of its bioaccessibility (Alegria, Garcia‐Llatas, and Cilla 2015). So, the samples were centrifuged at 4500 rpm for 60 min at 10°C and the supernatants were transferred into new amber tubes, flushed with N_2_, and stored at −20°C until extraction (Sriwichai et al. 2016).

### 2.6 Estimation of the Contribution of Each Single Cooked Crop and of the Porridges Prepared With Mixed Flours to Covering Child Nutrition Requirements

The coverage (%) of daily iron, zinc, and vitamin A requirements by foods (150 g) was calculated by taking into account the measured dialyzability (for iron and zinc) and bioaccessibility (for vitamin A) of micronutrients, as well as the recommended dietary allowances (RDA) and recommended safe intake (RSI for vitamin A) of children aged 6–23 months (FAO and WHO 2004). Calculations were done as follows:

$$\begin{array}{c} \text{Iron or zinc coverage}\;\left(\%\right)\\ =\text{Dialyzable iron or zinc}\;\left(\mathrm{mg}/150\;g\;\mathrm{DM}\right)\times 100/\text{iron or zinc}\;\mathrm{RDA} \end{array}$$

where the iron RDA = 5.8 mg/day and the zn RDA = 4.1 mg/day.

For vitamin A, a 50% conversion of the bioaccessible β‐carotene into retinol was applied (Bechoff et al. 2011; Dhuique‐Mayer et al. 2018).

$$\begin{array}{c} \text{Vitamin}\;A\;\text{coverage}\;\left(\%\right)\\ =\left(\text{Bioaccessible}\;\beta -\text{carotene}\;\left(\mathrm{\mu g}/150\;g\;\mathrm{FM}\right)/6\right)\times 100/\text{vitamin}\;A\;\mathrm{RSI} \end{array}$$

Vitamin A RSI = 400 μg RAE (Retinol Activity Equivalent).

### 2.7 Statistical Analysis

Data were computed using Microsoft Excel 2016 and Stata SE 16. Results are presented as mean ± SD and percentages. The normal distribution and homogeneity of variances for all variables were first checked using Shapiro–Wilk and Bartlett's tests. Differences in means between non‐biofortified and biofortified millet flours and porridges were compared using a Student's *t*‐test. The samples of cowpea, OFSP, and PBFs were compared using univariate analysis (ANOVA). These tests were used in the case of normal distribution and homogeneity of variances. Otherwise, nonparametric (Kruskal–Wallis, Wilcoxon–Mann–Whitney) tests were used for comparison. All the analyses were performed at the 5% significance level.

## 3 Results

### 3.1 Characteristics of Raw Materials

DM content was similar in biofortified and non‐biofortified millet and cowpea, regardless of the biofortification strategy used (*p* > 0.05) (Table 1). The dry matter content of OFSP increased by 9 mg/100 g DM in the sample biofortified with PL (*p* < 0.05) compared to its counterpart biofortified with PL + EM + MF. Inversely, in mixed flours, DM content was higher in the sample biofortified with PL + EM + MF (100%) than in the other samples (*p* < 0.05).

**TABLE 1:** Characteristics of raw materials (*n* = 11, each sample was measured in triplicate).

	Dry matter (mg/100 g)	Iron (mg/100 g DM)	Zinc (mg/100 g DM)	β‐carotene (mg/100 g DM)
*Millet flours*				
Non‐biofortified (M)	91.65 ± 0.34	2.58 ± 0.32	8.60 ± 0.88	—
Biofortified with cow dung CD (MB)	90.66 ± 0.08	2.40 ± 0.34	8.76 ± 0.12	—
*Cowpea*				
Non‐biofortified	86.57 ± 2.37	4.91 ± 0.96*	6.17 ± 0.63	—
Biofortified with poultry litter (PL)	88.96 ± 0.34	10.32 ± 0.92**	4.68 ± 0.06	—
Biofortified with PL + EM + MF	89.78 ± 0.20	12.76 ± 2.54**	4.50 ± 1.38	—
*Orange‐fleshed sweet potato (OFSP)*				
Non‐biofortified	28.37 ± 0.59*	1.64 ± 0.07*	1.35 ± 0.05*	58.04 ± 9.09
Biofortified with poultry litter (PL)	37.61 ± 0.72**	4.68 ± 1.45**	2.13 ± 0.30*	73.76 ± 18.12
Biofortified with PL + EM + MF	23.68 ± 0.18*	5.08 ± 1.52**	3.84 ± 0.93**	76.98 ± 9.73
*Mixed or blended flours*				
Non‐biofortified	90.48 ± 0.21*	4.73 ± 0.06	10.46 ± 0.37	12.76 ± 0.66*
Biofortified with CD + PL	90.16 ± 0.11*	5.09 ± 0.17	8.76 ± 0.52	12.04 ± 0.31*
Biofortified with CD + PL + EM + MF	100.33 ± 0.47**	5.06 ± 0.39	8.32 ± 1.20	21.71 ± 2.25**

*Note:* Results are presented as mean ± standard deviation (dry matter or DM).

Abbreviations: —, not measured; EM, efficient microorganisms; MF, mycorrhizal fungi.

* and **Significant difference in DM, iron, zinc, and β‐carotene between the same type of food sample in each column, ANOVA and Student's *t*‐tests and/or nonparametric tests (*p* < 0.05).

Biofortification with CD did not affect the iron and zinc content of millet. The two types of biofortification (PL and PL + EM + MF) efficiently increased the iron content of cowpea by two‐ to threefold, respectively. However, this was not the case for zinc, regardless of the biofortification strategy used (*p* > 0.05). Mineral contents in raw OFSP were differently affected by the two biofortification strategies: iron content was three times higher in OFSP biofortified with PL than in the non‐biofortified counterpart, whereas zinc content was not affected. Conversely, OFSP biofortified with PL + EM + MF had higher concentrations of iron (5.11 ± 1.52 mg/100 DM) and zinc (3.84 ± 0.93 mg/100 DM) than the non‐biofortified OFSP (1.64 ± 0.07 mg/100 DM for iron; 1.35 ± 0.05 mg/100 DM for zinc). β‐Carotene content was not significantly affected by biofortification (*p* > 0.05). The highest concentration of β‐carotene in blended flours was that made from biofortified millet, cowpea, and OFSP biofortified with PL + EM + MF (*p* < 0.05) compared to its counterparts.

### 3.2 Bioaccessibility and Dialyzability of Iron in Porridges Made With a Single Crop and in Porridges Made With Mixed Flours

Total iron content was similar in biofortified and non‐biofortified millet porridges (Table 2). The bioaccessible fraction (BF%) of iron was 23% and 25% for non‐biofortified and biofortified millet porridges, respectively. More than 70% of the iron was insoluble in both porridges.

**TABLE 2:** Bioaccessibility and dialyzability of iron in individual cooked crops and in the porridges prepared with mixed flours (*n* = 8).

	Total iron (mg/100 g)	Dialyzable (mg/100 g)	Bioaccessible (%)	Dialyzable (%)	SND (%)	Insoluble (%)
*Millet porridges*						
M	2.30 ± 0.01	0.24 ± 0.16	23	4	19	77
MB	2.68 ± 0.16	0.14 ± 0.12	25	1	24	75
*Boiled cowpea*						
BC	5.44 ± 0.73*	0.07 ± 0.00*	55	9**	46	45
BC1	5.14 ± 0.65*	0.40 ± 0.02*	43	29*	14	57
BC2	2.89 ± 0.06**	0.16 ± 0.01*	54	27*	29	46
*Porridges made with blended flours (PBFs)*						
PBF	2.86 ± 2.49	0.31 ± 0.15	29	14	15	71
PBF1	3.32 ± 0.92	0.53 ± 0.40	56	14	42	44
PBF2	4.53 ± 0.41	0.59 ± 0.39	41	13	28	59

*Note:* Results are presented as mean ± standard deviation (dry matter) and as percentage (%).

Abbreviations: BC, non‐biofortified boiled cowpea; BC1, boiled cowpea biofortified with poultry litter (PL); BC2, boiled cowpea biofortified with PL + EM + MF; M, porridge made with non‐biofortified millet flour; MB, porridge made with millet flour biofortified with cow dung (CD); PBF, porridge made with non‐biofortified blended flour; PBF1, porridge made with BF1; PBF2, porridge made with BF2; SND, soluble non‐dialyzable.

* and **Significant differences between the same type of food sample in each column, ANOVA and Student's *t*‐tests and/or nonparametric tests (*p* < 0.05).

Total iron content in boiled cowpea was similar in the non‐biofortified (BC) and biofortified (BC1) samples, but, surprisingly, decreased by 49% in biofortified sample BC2 (*p* < 0.05). Despite the low initial concentration of iron in BC2, dialyzable or approximate bioavailable iron was significantly higher in the biofortified cowpeas (27%–29%) than in the non‐biofortified counterpart (9%). The insoluble fraction of iron ranged between 45% and 57% in cowpeas with or without the use of ORP. In PBFs, total and bioaccessible iron were similar in the samples regardless of the biofortification strategy used (*p* > 0.05). Dialyzable iron was less than 15% in all samples (*p* > 0.05).

### 3.3 Bioaccessibility and Dialyzability of Zinc in Porridges Made With a Single Crop and in Porridges Made With Mixed Flours

Total zinc content was similar in biofortified and non‐biofortified millet porridges (Table 3). Surprisingly, the average concentration of zinc in the two samples prepared using biofortified cowpea decreased from 28% (BC1) to 61% (BC2) and was significantly lower than that of its non‐biofortified counterpart BC (*p* < 0.05).

**TABLE 3:** Bioaccessibility and dialyzability of zinc in individual cooked crops and in the porridges prepared with mixed flours (*n* = 8).

	Total zinc (mg/100 g)	Dialyzable (mg/100 g)	Bioaccessible (%)	Dialyzable (%)	SND (%)	Insoluble (%)
*Millet porridges*						
M	2.57 ± 0.12	0.66 ± 0.09	62	34	28	38
MB	2.53 ± 0.10	0.80 ± 0.40	60	34	26	40
*Boiled cowpea*						
BC	4.67 ± 0.18*	1.60 ± 0.13**	69	56	13	31
BC1	3.33 ± 0.46*	0.87 ± 0.13*	70	41	29	30
BC2	1.79 ± 0.02**	0.91 ± 0.06*	71	54	17	29
*Porridges made with blended flours (PBFs)*						
PBF	3.64 ± 0.23**	0.66 ± 0.06	43	25	18	57
PBF1	1.76 ± 0.67*	0.50 ± 0.11	39	23	16	61
PBF2	2.57 ± 0.16**	0.74 ± 0.69	43	31	12	57

*Note:* Results are presented as mean ± standard deviation (dry matter) and as percentage (%).

Abbreviations: BC, non‐biofortified boiled cowpea; BC1, boiled cowpea biofortified with poultry litter (PL); BC2, boiled cowpea biofortified with PL + EM + MF; M, porridge made with non‐biofortified millet flour; MB, porridge made with millet flour biofortified with cow dung (CD); PBF, porridge made with non‐biofortified blended flour; PBF1, porridge made with BF1; PBF2, porridge made with BF2; SND, soluble non‐dialyzable.

* and **Significant differences between the same type of food sample in each column, ANOVA and Student's *t*‐tests and/or nonparametric tests (*p* < 0.05).

Similar results were obtained for PBFs, where total zinc lowered by 51% in PBF1 and 29% in PBF2. Dialyzable zinc was higher in BC than in BC1 and in BC2 (*p* < 0.05). More than 60% of zinc was bioaccessible in millet porridges and in boiled cowpeas, while less than 50% of zinc was bioaccessible in PBFs. No difference was observed between bioaccessible, soluble non‐dialyzable, and insoluble zinc in all the food samples, regardless of the biofortification system used (*p* > 0.05).

### 3.4 Bioaccessibility of β‐Carotene in Mashed OFSP and in Porridges Made With Mixed Flours

Unlike in the raw materials, the total concentration of β‐carotene in cooked biofortified OFSP (OFSP1 and OFSP2) represents around 58%–59% of that of the non‐biofortified OFSP, with no difference between samples (*p* < 0.05). But surprisingly, overall bioaccessibility was two to three times better in biofortified OFSP than in the non‐biofortified counterpart. In the porridges made with the mixed flours or PBFs, the use of biofortified crops significantly increased the concentration of β‐carotene from 8 to 30 mg/100 g FW for PBF1 and PBF2, respectively. However, overall β‐carotene bioaccessibility was similar in all food samples (Table 4).

**TABLE 4:** β‐Carotene content and bioaccessibility in individual cooked crops and in the porridges prepared with mixed flours (*n* = 6).

	Total β‐carotene (mg/100 g FM)	β‐carotene bioaccessibility	
		mg/100 g FM	%
*Porridges made with blended flours (PBFs)*			
PBF	15.26 ± 1.58*	1.41 ± 0.40	8.1
PBF1	24.14 ± 4.95*	1.34 ± 0.30	7.1
PBF2	45.72 ± 6.80**	1.68 ± 0.21	7.8
*Mashed OFSP*			
OFSP	86.77 ± 3.46**	1.22 ± 0.06*	1.4*
OFSP1	50.72 ± 3.87*	1.24 ± 0.01*	2.5*
OFSP2	51.83 ± 10.03*	2.08 ± 0.46**	4.1**

*Note:* Results are presented as means ± standard deviation (fresh weight—FW) and as percentages (%).

Abbreviations: OFSP, mash made with non‐biofortified OFSP; OFSP1, mash made with OFSP biofortified with PL; OFSP2, mash made with OFSP fertilized with PL + EM + MF; PBF, porridge made with blended flour containing non‐biofortified millet, cowpea, and OFSP; PBF1, porridge made with BF1; PBF2, porridge made with BF2.

* and **Significant differences between the same type of food sample in each column, ANOVA and/or nonparametric tests (*p* < 0.05).

### 3.5 Contribution of Porridges Made From a Single Crop and Porridges Made With Mixed Flours to Covering Child Nutrition Requirements

**FIGURE 1:**
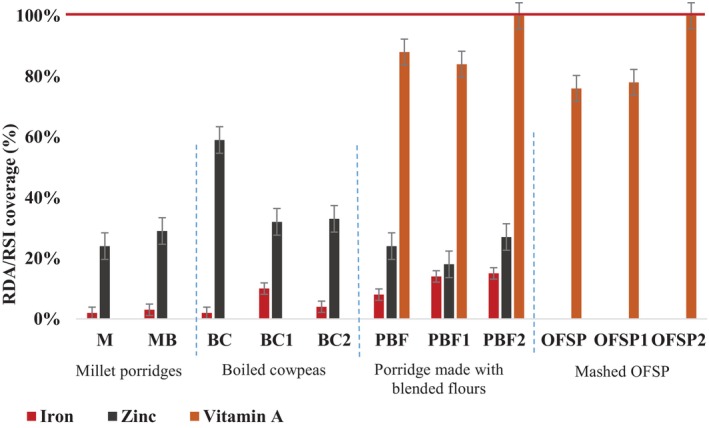
Coverage (%) of iron, zinc, and vitamin A requirements of children 6–23 months old by complementary foods, considering their bioaccessibility and dialyzability. M: Porridge made with non‐biofortified millet flour; MB: Porridge made with millet flour biofortified with cow dung (CD); BC: Non‐biofortified boiled cowpea, BC1: Boiled cowpea biofortified with poultry litter (PL), BC2: Boiled cowpea biofortified with PL + EM + MF. PBF: Porridge made with non‐biofortified blended flour, PBF1: Porridge made with BF1, PBF2: Porridge made with BF2. OFSP: Mashed made with non‐biofortified OFSP; OFSP1: Mashed made with OFSP biofortified with PL; OFSP2: Mashed made with OFSP fertilized with PL + EM + MF.

The consumption of millet porridge (150 g) would provide a very low coverage of iron requirements (< 5%) with or without biofortification (Figure 1). The consumption of boiled cowpeas prepared using BC1 would cover up to 10%, slightly better than using non‐biofortified cowpeas (2%). The results for porridges made with mixed flours were even better, as up to 15% of the iron requirement might be covered if biofortified foods are used together.

Regarding zinc requirements, the consumption of 150 g of food samples would cover less than 40% of children's RDA of children except for the non‐biofortified boiled cowpea, which would cover more than 50% of the requirements. Overall, biofortification would not improve zinc intake based on the consumption of any food type.

Regarding vitamin A, considering bioaccessibility and applying a 50% conversion to retinol, the percentage coverage of the RSI for children would be up to 70% and 80% with the consumption of OFSP/OFSP1 and PBF/PBF1, respectively. However, the consumption of 150 g of OFSP2 and PBF2 foods would cover 100% of the children's RSI.

## 4 Discussion

The objective of this study was to assess the effects of different agronomic biofortification strategies involving the use of ORPs, namely PL, CD alone, or in combination with EM and MF on the bioaccessibility of iron, zinc, and β‐carotene in local complementary foods made from millet, cowpea, and OFSP. In the present study, we have shown that these biofortification strategies are able to increase the concentrations of some micronutrients, but not in all cases. For example, we have shown that the use of cow dung alone as a fertilizer does not seem to affect the concentration of iron and zinc in millet, as similar concentrations were found in biofortified and non‐biofortified millet flours and porridges. On the contrary, the biofortification strategy using PL alone or as a combination of PL + EM + MF had an impact on the iron content of raw cowpea and OFSP. The twofold higher iron content in cowpea and OFSP biofortified with PL + EM + MF may be linked to the beneficial effect of MF, which are known to promote better uptake and bioavailability of some minerals in host plants (Miransari 2013; Cao, Wang, and Huang 2015; Mustafa et al. 2016; Mei et al. 2019; Khan, Shah, and Tian 2022; Teklu et al. 2023). The significant amounts of iron and zinc found in OFSP after biofortification make the promotion and consumption of this staple food more advantageous.

When each crop was cooked separately or when porridges were prepared using a mixture of all crops, a decrease in iron was observed in one of the biofortified cowpea food samples, and a decrease in zinc was observed in cowpea and in the mixture of PBFs. The decrease in iron (from 12.7 to 2.89 mg) and in zinc (4.5–1.79 mg) in cowpea observed after boiling in this study could be explained by the effect of washing and soaking, which eliminates exogenous iron and zinc from different sources of contamination. In fact, several authors have reported that the washing and soaking stages can reduce mineral contents, due to leaching into the soaking water (Lestienne et al. 2005; Platel, Eipeson, and Srinivasan 2010; Afify et al. 2011; Akissoé et al. 2020). In the present study, cowpeas were soaked for about 14 h before being boiled. Despite these disappointing results, an improvement in iron dialyzability was observed in the case of cowpea biofortification. This suggests that the use of PL or the mixture of PL + EM + MF as a fertilizer increases the dialyzability of iron in cowpea. The values of dialyzable iron in biofortified cowpea found in this study (27%–29%) are higher than those reported by Coelho in biofortified cowpea cultivars (3.3%–3.8%) consumed in Brazil (Coelho et al. 2021). This confirms the relevance of the abovementioned biofortification strategies developed in the OR4FOOD project on iron dialyzability. Apart from the impact of agro‐biofortification, several other factors, including the food matrix, food processing, texture, and the presence of inhibitors or enhancers, may explain mineral contents and their release after in vitro digestion (Mouquet‐Rivier et al. 2008; Zou et al. 2014; Icard‐Vernière et al. 2016; Rousseau et al. 2019; Sulaiman, Givens, and Anitha 2021). Another possible explanation is the interaction between iron and zinc (Meyer et al. 2013; Coelho et al. 2021). In the study by Meyer et al. (2013), the dialyzable fraction of iron was found to be 3.1 times lower than that of zinc. Also, according to Muleya, Young, and Bailey (2021), the amount of iron and zinc in reagents (enzymes) may represent at least 50% of the total iron/zinc in the final digestion mixture, which may explain the higher bioaccessible fractions of iron and zinc found in our study compared to the results of other studies.

Regarding β‐carotene measurements, the significantly higher concentration of β‐carotene found in PBF2 biofortified with the mixture of PL + EM + MF could be linked to the high dry matter (100%) of this sample compared to the other samples (around 90%). Millet and cowpea also contain β‐carotene, which concentration vary depending on the varieties, but also on the cultivation conditions (Fairweather‐Tait et al. 2007; FAO/INFOODS 2019). Even if literature is scarce about the effect of biofortification on β‐carotene content of these two crops, it cannot be excluded that the use of the crops grown with the mixture of PL + EM + MF was able to increase their β‐carotene content, and thus the one of the porridges prepared with the mixed flour. The investigation of the effect of biofortification on β‐carotene of millet and cowpea, would be a plus. However, the average amounts of β‐carotene found in all our OFSP samples were higher than those reported by Sylla et al. (2020) for three OFSP varieties (Kandee, Caromex 1 and Caromex 2) introduced in the groundnut basin of Senegal in 2015 to fight against vitamin A deficiency, meaning the screening carried out in the OR4FOOD project was efficient. The higher β‐carotene content in mashed OFSP compared to the concentrations reported by Badiane (2018) and by Bechoff et al. (2011), could be due to the different OFSP varieties used as well as to the cooking methods. In our study, OFSP were steamed, whereas in the abovementioned studies, they were boiled before being mashed or processed into other complementary foods.

The use of PL, EM, and MF doubled the bioaccessible β‐carotene in mashed OFSP. In fact, after in vitro digestion, the bioaccessible β‐carotene in OFSP2 (4.1%) was three times higher than in OFSP (1.4%) and almost twice that in OFSP1 (2.5%). This confirms Khan's hypothesis that the combination of arbuscular MF and bacteria (EM) plays a positive role in nutrient uptake and availability through mycorrhization (Khan et al. 2023). According to Khan et al. (2020), arbuscular MF are one of the most important microbiota involved in a symbiotic relationship with plant roots. Several authors reported an increase in photosynthetic activities, nutrient uptake, plant growth, as well as improved drought tolerance in some plants after inoculation with arbuscular MF (Gernns, Alten, and Poehling 2001; Achatz et al. 2010; Borde, Dudhane, and Jite 2011; Shamshiri and Fattahi 2016; Meddich et al. 2021; Khan, Shah, and Tian 2022). Bioaccessible β‐carotene contents ranging from 7.1% to 8.1% were found in PBFs with no significant difference between samples. These results are close to those reported by Bechoff et al. (2011) in porridge containing OFSP (9.9%). The bioaccessible β‐carotene found in the present study for both PBFs and mashed OFSP may be linked to the addition of oil after cooking, as is the case for many homemade complementary foods (porridge, puree) given to children and promoted during nutrition education sessions in Senegal. Indeed, like other investigators, Bechoff et al. observed that, the addition of oil prior or during cooking increased the incorporation of β‐carotene into micelles whereas adding oil after cooking did not (Bengtsson et al. 2010; Bechoff et al. 2011; Dhuique‐Mayer et al. 2018). However, physical barriers (food matrix) could limit β‐carotene bioaccessibility as observed by Sriwichai et al. (2016), who reported bioaccessible β‐carotene values < 1% in a wide range of fresh leaves despite the addition of 80 μL colza oil prior to in vitro digestion.

In terms of coverage of nutrient requirements by the complementary foods based on measured micronutrient dialyzability or bioaccessibility, consumption of 150 g of mashed OFSP2 and PBF2 covered the total (100%) vitamin A requirement of children aged 6–23 months. All percentages of coverage found in this study for both porridges (88%, 84%, 100%) and mashed OFSP (76%, 78%; 100%) were higher than those reported by Bechoff et al. (2011), Ekessa et al. (2012), and Dhuique‐Mayer et al. (2018) for porridge made from blended flour containing OFSP, fortified with *moisilongo* palm oil, and for processed baby food. Our results for vitamin A requirements were in the range of the coverage (%) found by Amoussa‐Hounkpatin et al. (2012) for a traditional leafy vegetable dish (71%–129%) composed of different ingredients, confirming the high quality of the OFSP variety used in the present study.

Regarding iron and zinc requirements, percentages of coverage less than < 20% (for iron) and < 50% (for zinc except for non‐biofortified cowpea) were found with the consumption of 150 g of complementary foods. These results suggest that the addition of other local foods (lipids and vitamin C rich foods) could optimize iron and zinc absorption. However, some of the possible interactions between iron and zinc, which may be the main reasons why biofortification did not work for zinc in millet, cowpea, and PBFs in this study, require further investigation. Even if biofortification only works to a limited extent for iron (in cowpea and PBFs), it could nevertheless be used as a complement to other approaches, because iron deficiency is a huge challenge. Healthy agronomic biofortification could play a role, even if only a small one, especially in the groundnut basin of Senegal, a region with limited access to animal‐based products.

## 5 Conclusion

Biofortification is increasingly being promoted as a low cost and sustainable approach for tackling micronutrient deficiencies. Using different approaches to increase populations' micronutrient intakes is essential if we are to achieve the sustainable nutrition goals by 2030. In the present study, the combination the most promising varieties of crops, together with the agronomic strategies of biofortification yielded successful results, particularly for β‐carotene and iron content and their bioaccessibility, although not for zinc. In addition, measuring in vitro bioaccessibility of the different biofortified crops highlighted the limitations of only measuring the concentration in the raw and the cooked material to estimate the fraction potentially available for absorption. These findings are promising for sustainable food–based solutions that diversify what we grow and eat to fight against micronutrient deficiencies in context of monotonous and low‐quality diets.

## Author Contributions

**Mbeugué Thiam:** conceptualization (lead), data curation (lead), formal analysis (lead), methodology (lead), validation (equal), writing – original draft (lead), writing – review and editing (lead). **Adama Diouf:** conceptualization (lead), funding acquisition (lead), methodology (equal), resources (equal), supervision (lead), validation (lead), writing – review and editing (equal). **Christèle Icard‐Vernière:** data curation (supporting), formal analysis (equal), methodology (supporting), validation (equal), writing – review and editing (supporting). **Sylvie Avallone:** data curation (equal), formal analysis (supporting), methodology (supporting), validation (equal), writing – review and editing (supporting). **Ndèye Fatou Ndiaye:** methodology (equal), resources (equal), validation (equal), writing – review and editing (supporting). **Marielle Atala De Souza:** formal analysis (equal), methodology (supporting), validation (equal). **Jean‐Michel Médoc:** funding acquisition (lead), project administration (lead), resources (supporting), validation (equal). **Nicole Idohou‐Dossou:** funding acquisition (lead), project administration (lead), resources (supporting), validation (lead), writing – review and editing (supporting). **Christèle Humblot:** conceptualization (lead), data curation (equal), funding acquisition (supporting), methodology (lead), resources (supporting), supervision (lead), validation (lead), writing – review and editing (equal).

## Ethics Statement

The authors have nothing to report.

## Conflicts of Interest

The authors declare no conflicts of interest.

## Data Availability

The data that support the findings of this study are available on request from the corresponding author. The data are not publicly available due to privacy or ethical restrictions.
